# Anthropometry, body composition and chronic disease risk factors among Zambian school-aged children who experienced severe malnutrition in early childhood

**DOI:** 10.1017/S0007114521003457

**Published:** 2022-08-14

**Authors:** Lackson Kasonka, Grace Munthali, Andrea Mary Rehman, Molly Chisenga, Samuel Wells, Jonathan C. K. Wells, Suzanne Filteau

**Affiliations:** 1 University Teaching Hospital, Women and Newborn, Lusaka, Zambia; 2 National Institute for Scientific and Industrial Research, Lusaka, Zambia; 3 Faculty of Epidemiology and Population Health, London School of Hygiene and Tropical Medicine, London, UK; 4 Faculty of Infectious and Tropical Diseases, London School of Hygiene and Tropical Medicine, London, UK; 5 Institute of Child Health, University College London, London, UK

**Keywords:** Severe acute malnutrition, Body composition, Chronic disease risk, School-aged children

## Abstract

There is limited information as to whether people who experience severe acute malnutrition (SAM) as young children are at increased risk of overweight, high body fat and associated chronic diseases in later life. We followed up, when aged 7–12 years, 100 Zambian children who were hospitalised for SAM before age 2 years and eighty-five neighbourhood controls who had never experienced SAM. We conducted detailed anthropometry, body composition assessment by bioelectrical impedance and deuterium dilution (D2O) and measured blood lipids, Hb and HbA1c. Groups were compared by linear regression following multiple imputation for missing variables. Children with prior SAM were slightly smaller than controls, but differences, controlling for age, sex, socio-economic status and HIV exposure or infection, were significant only for hip circumference, suprailiac skinfold and fat-free mass index by D2O. Blood lipids and HbA1c did not differ between groups, but Hb was lower by 7·8 (95 % CI 0·8, 14·7) g/l and systolic blood pressure was 3·4 (95 % CI 0·4, 6·4) mmHg higher among the prior SAM group. Both anaemia and high HbA1c were common among both groups, indicating a population at risk for the double burden of over- and undernutrition and associated infectious and chronic diseases. The prior SAM children may have been at slightly greater risk than the controls; this was of little clinical significance at this young age, but the children should be followed when older and chronic diseases manifest.

Zambia, as well as other countries in Sub-Saharan Africa, has improved both economic and many health indicators in recent years and is in the midst of the nutritional and epidemiological transition. While the prevalence of low weight-for-age and severe acute malnutrition (SAM) remains high^(1,2)^, there is also increasing prevalence of overweight and obesity, resulting in a ‘double burden’ of malnutrition^(3)^. Furthermore, early undernutrition, if followed by later overweight due to biological or social reasons, may increase the risk of non-communicable chronic diseases (NCD)^(4,5)^. For example, a history of childhood malnutrition was a risk factor for diabetes among Ethiopian adults^(6)^; insulin production and glucose tolerance may remain abnormal in anorexia nervosa patients even after nutritional recovery^(7)^; and exposure to famine during childhood was associated with increased adult diabetes in the Netherlands^(8)^ and China^(9)^. In the study from China, a higher BMI or consumption in adulthood of a typically Western dietary pattern, rather than the traditional diet, increased the risk of hyperglycaemia^(9)^.

HIV infection, for which prevalence is high in Zambia^(1)^, may lead to malnutrition. An audit from University Teaching Hospital, Lusaka, showed that the prevalence of HIV infection among children on the ward was higher than maternal antenatal prevalence at the time^(2)^. Among Tanzanian adults, it appeared that prior malnutrition associated with HIV infection was a risk for later low insulin production^(10)^. Therefore, HIV should be considered as an associated factor when investigating long-term follow-up of African children with SAM.

Given the rising prevalence of overweight and NCD in areas of Africa where HIV and SAM remain prevalent, it is important to evaluate the separate and any interacting associations of exposure to HIV or SAM in early life with later risks of NCD. While overt NCD typically emerge in adult life, many studies have linked markers of fetal undernutrition with markers of poorer cardiometabolic health during childhood, such as raised blood pressure, insulin resistance and dyslipidaemia that may track into adulthood^(11,12)^. Moreover, these metabolic traits have been linked with catch-up in weight and its association with elevated levels of body fat^(13,14)^. Whether these associations are evident in children in low-income settings who have recovered from postnatal SAM remains unclear. The aim of the present study was to determine whether SAM in early childhood increased risk factors for chronic diseases in later childhood and whether HIV infection or exposure modified this risk. We recruited children who were hospitalised in University Teaching Hospital, Lusaka, several years previously when under 2 years of age and compared their anthropometry, body composition and NCD risk factors with those of not-previously malnourished neighbourhood controls. While children rarely have clinically detectable NCD, it is important to determine elevated risk factors at a young age when interventions can be started before clinical problems develop.

## Methods

### Study design

The study was a cohort study which traced children who had previously been hospitalised for SAM. Controls were children who had never experienced SAM and were recruited when we followed up the prior SAM children.

### Participants

Children were recruited based on hospital records that they had been admitted when less than 2 years old to the University Teaching Hospital, Lusaka, paediatric nutrition ward for treatment of SAM between 2010 and 2014. Although records were available in the malnutrition ward for most children admitted, records were sometimes missing for children who died on the ward, where mortality is high^(2)^, or who were transferred to another ward after recovery from SAM but before discharge from the hospital. Records from the most recent dates were used first for recruitment as it was expected that these families were less likely to have changed their contact details; indeed, many of the earlier files were missing contact details such as mobile phone numbers. Efforts were made to contact those with contact details but in many cases addresses or phone numbers had changed. The malnutrition clinic followed WHO guidelines for treating SAM^(15)^, starting all children on F75 and, once they were stabilised and beginning to recover, moving them on to ready-to-use therapeutic foods, either in hospital or as outpatients.

Recruited children and families were asked to recommend a friend of similar age and preferably sex, whose family could be approached for inclusion as controls. Frequency matching of exposed children to unexposed community controls was used to reduce the variability in outcomes due to these potential confounding factors. To do this, we recruited controls from the same neighbourhoods as prior SAM children and aimed for a similar age and sex profile in the two groups overall. Other than age, sex and neighbourhood, the only additional inclusion criterion for control children was that they had never been hospitalised for SAM, according to parental report.

### Ethics

This study was conducted according to the guidelines laid down in the Declaration of Helsinki, and all procedures involving human participants were approved by the University of Zambia Biomedical Research Ethics Committee and the London School of Hygiene and Tropical Medicine Ethics Committee. Written consent was obtained from carers. Written or verbal assent was also obtained from all children. If severe anaemia, high HbA1c, or other reason for medical intervention was found on clinical examination, children were referred to the local government services on the same site as the research clinic.

### Assessments at study visits

Children and parents or guardians were invited to the University Teaching Hospital research clinic for a single visit. Questionnaires were used to collect demographic, socio-economic and morbidity history data and children were given a clinical examination. We collected data on history of hospitalisation to determine if community control children had ever been hospitalised for SAM. We asked girls if they had started menstruating and we examined boys for Tanner stage.

Anthropometry data – weight, height, mid-upper arm circumference, waist and hip circumferences, triceps, subscapular and suprailiac skinfolds – were collected by experienced anthropometrists. Blood pressure was measured using an automatic Omron IP20 sphygmomanometer. Grip strength was measured using a Takei GRIP-D dynamometer. The measurements, excluding body composition, were taken twice and analyses used the mean for most variables but the maximum for grip strength. Finger-prick blood samples were used for measurement of Hb and HbA1c using handheld instruments from Hemocue; due to problems with equipment or supplies, some children did not have these measurements. HbA1c was the only feasible method for assessing glucose tolerance in the study because it was not possible to have children come to the clinic fasting. Venous blood samples were collected for triglycerides and total, HDL- and LDL-cholesterol. Blood lipids were measured using enzymatic assay kits from Pointe Scientific.

Body composition was measured using two independent methods: bioelectrical impedance (BIA) using Tanita BC418 instrumentation and deuterium dilution (D2O) according to standard methods^(16)^. For BIA, fat mass and fat-free mass (FFM) were obtained using manufacturers’ equations that take into account weight, age and sex. For D2O dilution, a baseline saliva sample was collected from participants at least 2 h after their last meal. Each participant then received an oral dose (0·1 g/kg body weight) of D2O (99·8 % atom excess, Cambridge Isotope Laboratories). Two end-point saliva samples were collected at 3 and 4 h after D2O dose ingestion; if they agreed within 3 mg/kg, indicating equilibration by 3 h, the average was used; if not, the 4-h sample was reanalysed as the duplicate. Saliva samples were stored in plastic saliva vials at –20°C until analysis for D2O abundance using the Fourier transform IR spectrometer (Agilent Technologies; model 4500 s). The enrichment was calculated by subtracting the value of the baseline sample from the value of the post-dose sample. The calculated D2O enrichment was then used in the calculation of body composition, using published values for FFM hydration to convert body water to FFM^(17)^. Fat mass was calculated by difference of weight and FFM.

### HIV status and exposure

All parents were asked about the child’s most recent HIV test and about the mother’s HIV status during pregnancy and breast-feeding. In some cases, child HIV status information was available from case medical files when children were in hospital with SAM. Children of HIV-uninfected mothers who had never been tested were included as HIV-unexposed, uninfected. Children of HIV-infected mothers who had not themselves been tested or if their status was missing were included as HIV-exposed, uninfected since we expected they would have begun to show symptoms on clinical examination if they were HIV-infected. For some children, maternal HIV was unknown or the mother did not wish to provide it; these children were coded as unknown HIV exposure.

### Data management and statistical analyses

Data were collected using the RedCap system and imported into Stata 16 for analysis. Height-for-age and BMI-for-age *Z*-scores were calculated using the WHO standards in the Stata zanthro command. We categorised anaemia using the WHO cut-offs for children 5–11 years since these were the majority; moderate anaemia was Hb 80–115 g/l and severe anaemia was Hb < 80 g/l. We considered HbA1c ≥ 6·5 % to indicate diabetes. Principal component analysis was used to create a socio-economic status (SES) score from questionnaire data on family assets – car, bicycle, radio, television, refrigerator, mobile phone, livestock and poultry. The SES score was divided into terciles of low, middle and high SES. The principal component analysis for SES was based on a larger group (*n* 514) of families from a wider cross-section of Lusaka neighbourhoods since we were conducting a related study in parallel. We collected data on both maternal and paternal education and occupation but present only maternal since a large proportion of paternal data was missing since many women were not living with a partner.

The main exposure variable was hospitalisation for SAM before 2 years of age. Outcome variables were anthropometry, fat mass index (FMI) and fat-free mass index (FFMI) by BIA and D2O (calculated as fat mass or FFM in kg/height in m^2^ as for BMI), blood lipids, blood pressure, Hb, HbA1c and grip strength. Variables related to fat, that is, FMI and skinfolds, were log-normally distributed so log-transformed variables were used in analyses and medians and inter-quartile ranges are presented. For two male participants, fat mass or FFM measured by BIA and D2O differed by ∼5 kg, suggesting an error, so their FMI and FFMI results were removed from analysis. To analyse all children’s records, including the seventy-five which were missing at least one outcome (including the two boys with discrepant body composition results) or exposure variable (Hb had highest percentage of missing data, 41/185 (22·2 %), other variables (HIV exposure, mother’s marital status, education and occupation, grip strength, body composition, HbA1c, blood pressure, blood lipids) had at most 10/185 (5·4 %) missing values), we used multiple imputation with chained equations to generate ten multiple imputation datasets including exposures (sex, age, previous SAM, HIV status, mother’s education, mother’s occupation and SES category) and all outcomes in the imputation model. The primary analyses, using linear regression, were controlled for age and sex. We then conducted multivariable analyses adjusting for factors which either differed between the prior SAM and control children or are known to influence the outcomes: HIV status (coded as HIV-infected, HIV-exposed, uninfected or HIV-unexposed, uninfected) and SES terciles^(18)^. Pubertal stage was not included as a covariate in analyses since most children were pre-pubertal and pubertal stage was collinear with age.

### Sample size

A sample size of 100 per group was chosen to permit detection, at 90 % power, of differences of 2 % fat between those previously malnourished and not, assuming 20 % body fat (sd 4) in the control children. This estimate was based on total body fat percentage and differences we found previously between HIV-exposed and HIV-exposed, uninfected children of a similar age to the current study children^(18)^ and our assumption that prior SAM would induce slightly larger differences. This number of children provided 90 % power to detect biologically relevant between-group differences in risk factor markers. In the end, we recruited 100 previously malnourished but only eighty-five not-previously malnourished controls for whom recruitment was slower than expected, in part because we had to work around children’s time in school and parental jobs.

## Results

Of 2034 children hospitalised for SAM in the years of interest, 2010–2014, for only 687 did the medical records have contact details. Of these 687, contact details for 530 seemed no longer valid so they could not be contacted. Of those we found, thirty did not wish to join the study, nine had moved out of Lusaka, three were not eligible (one due to severe disability, two because they did not come with parents or guardians who could sign consent forms) and fifteen were not included because we had already achieved the planned sample size (online Supplementary Fig. S1.)

The study groups contained approximately equal numbers of girls and boys (Table 1). The mean age of the control children was 1·4 years higher than that of the children with prior SAM. Consequently, more control boys were at higher Tanner stage and two control girls, compared with no prior SAM girls, had started menstruating; nevertheless, both groups were mainly pre-pubertal. A sizeable proportion of children in both groups were either HIV-exposed or infected. About half the children in each group indicated they had experienced illness in the past month, but the vast majority of these were colds and no serious illness was reported (data not shown). Maternal variables did not differ between groups. SES based on an asset score was slightly higher for the prior SAM children; however, it is notable that SES was low in both groups compared with the larger population which contributed data to the principal component analysis.

**Table 1.** Characteristics of children according to whether or not they had previously experienced SAM (Numbers and percentages; mean values and standard deviation)

	SAM		No SAM		
	*n*	%	*n*	%	*P*
*n*	100		85		
Sex, male	51	51	40	47	0·59
Age (years)					
Mean	8·5		9·9		< 0·001
sd	0·7		1·8		
HIV exposure					0·13
Unexposed	69	69	64	75	
Exposed, uninfected	24	24	13	15	
Infected	4	4	1	1	
Unknown exposure	3	3	7	8	
Girls started menstruating	0/49	0	2/45	4	0·14
Boys’ Tanner stage					0·002
1	35/51	69	14/40	35	
2	16/51	31	19/40	48	
3	0	0	5/40	13	
4	0	0	2/40	5	
Mother’s marital status					
Married/cohabiting	78	78	66	78	0·94
Single	7	7	7	8	
Widowed	6	6	3	4	
Divorced	8	8	8	9	
Unknown	1	1	1	1	
Mother’s education					
Primary or less	45	45	34	40	0·67
Some secondary	39	39	30	35	
Completed secondary	8	8	11	13	
College, university	7	7	8	9	
Unknown	1	1	2	2	
Mother’s occupation					
Employed	57	57	53	62	0·61
Housewife	38	38	31	36	
Unemployed	2	2	0	0	
Other/unknown	3	3	1	1	
Socio-economic tertiles*					0·002
Low	69	69	72	85	
Medium	12	12	6	7	
High	19	19	7	8	

HEU, HIV-exposed, uninfected; HUU, HIV-unexposed, uninfected; SAM, severe acute malnutrition.

* Calculated using principal components analysis from a list of assets.

All correlations among the different indicators of body composition – FMI and FFMI by BIA and D2O and triceps, subscapular and suprailiac skinfold thicknesses – were significant at *P* < 0·001. Correlation coefficients among skinfolds ranged from 0·83 to 0·92; all correlated with FMI by BIA with coefficients of 0·74–0·78 and with FMI by D2O with coefficients of 0·59–0·62. Correlation coefficients for FFMI by either method with skinfolds were fairly low: 0·43–0·54 for FFMI by BIA and 0·27–0·38 for FFMI by D2O.

Comparisons of outcomes between previously malnourished children and controls following multiple imputation of missing values are shown in Tables 2 and 3, and the results without multiple imputation are shown in online Supplementary Tables S1 and S2. Results were comparable for these two sets of analyses. Based on *Z* scores for height and BMI, the children were slightly smaller than the WHO reference population, but only sixteen (16 %) of the prior SAM children and eight (9 %) of the control children had height *Z* score <–2. BMI *Z* score <–2 was seen for eleven (11 %) of the prior SAM children and seven (8 %) of the control children while two children in each group had BMI *Z* score > 1, indicating overweight. Although, when controlled for age and sex, children with prior SAM were slightly smaller, as indicated by the consistent negative coefficients, few differences reached statistical significance – only hip circumference, subscapular and suprailiac skinfolds, FMI by BIA and FFMI by D2O – with a borderline difference for weight (Table 2). Adjusting for HIV exposure or infection and SES had minimal effects on the coefficients; statistically significant differences remained for hip circumference, suprailiac skinfold and FFMI by D2O.

**Table 2.** Association of prior exposure to SAM with anthropometry, body composition and grip strength using data from multiple imputation,* (Numbers; 95 % confidence intervals)

Outcome	*n*	Imputed marginal means†	95 % CI	Difference adjusted for age and sex	95 % CI	*P*	Multivariable coefficient‡	95 % CI	*P*
Height (cm)									
No SAM	85	129·1	127·1, 131·1	–1·02	–3·12, 1·08	0·34	–1·21	–3·43, 1·01	0·28
SAM	100	128·1	126·1, 130·1						
Height-for-age *Z*									
No SAM	85	–0·73	–0·97, −0·49	–0·15	–0·50, 0·20	0·4	–0·18	–0·55, 0·19	0·34
SAM	100	–0·88	–1·10, −0·65						
Weight (kg)									
No SAM	85	26·6	25·2, 28·0	–1·40	–2·83, 0·04	0·06	–1·50	–3·01, 0·01	0·05
SAM	100	25·2	24·1, 26·3						
BMI (kg/m^2^)									
No SAM	85	15·6	15·1, 16·1						
SAM	100	15·2	14·8, 15·5	–0·46	–1·03, 0·11	0·12	–0·48	–1·15, 0·19	0·16
BMI-for-age *Z*									
No SAM	85	–0·57	–0·79, −0·34						
SAM	100	–0·78	–1·00, −0·57	–0·22	–0·54, 0·10	0·18	–0·21	–0·55, 0·14	0·24
MUAC (cm)									
No SAM	85	18·6	18·1, 19·1	–0. 5	–1·0, 0·1	0·12	–0·5	–1·1, 0·2	0·15
SAM	100	18·1	17·8, 18·5						
Hip circumference (cm)									
No SAM	85	66·7	64·8, 68·5						
SAM	100	64·4	63·1, 65·6	–2·3	–4·3, −0·3	0·03	–2·4	–4·6, −0·3	0·03
Waist circumference (cm)									
No SAM	85	55·4	54·2, 56·5						
SAM	100	54·6	53·8, 55·8	–0·8	–2·0, 0·5	0·23	–0·8	–2·2, 0·6	0·24
Triceps skinfold (mm)§									
No SAM	85	7·7	7·2, 8·3						
SAM	100	7·2	6·8, 7·6	–0·03	–0·07, 0·01	0·09	–0·03	–0·07, 0·01	0·14
Subscapular skinfold (mm)§									
No SAM	85	6·2	5·8, 6·6						
SAM	100	5·7	5·5, 6·0	–0·04	–0·07, −0·00	0·03	–0·03	–0·07, 0·00	0·08
Suprailiac skinfold (mm)§									
No SAM	85	5·1	4·7, 5·5						
SAM	100	4·6	4·3, 4·8	–0·05	–0·08, −0·01	0·02	–0·04	–0·09, 0·0	0·05
FMI by BIA (kg/m^2^)§									
No SAM	85	2·9	2·7, 3·1						
SAM	100	2·6	2·5, 2·8	–0·04	–0·07, −0·00	0·05	–0·04	–0·08, 0·00	0·07
FMI by D2O (kg/m^2^)§									
No SAM	85	2·5	2·2, 2·8						
SAM	100	2·6	2·4, 2·8	0·01	–0·05, 0·08	0·65	0·01	–0·06, 0·08	0·79
FFMI by BIA (kg/m^2^)									
No SAM	85	12·5	12·2, 12·8						
SAM	100	12·3	12·1, 12·6	–0·2	–0·5, 0·2	0·32	–0·2	–0·5, 0·2	0·39
FFMI by D2O (kg/m^2^)									
No SAM	85	12·8	12·6, 13·1						
SAM	100	12·3	12·1, 12·6	–0·5	–0·9, −0·1	0·02	–0·5	–0·9, −0·0	0·03
Grip strength (kg)									
No SAM	85	13·1	12·4, 13·9	–0·7	–1·6, 0·1	0·1	–0·6	–1·6, 0·3	0·17
SAM	100	12·4	11·7, 13·0						

BIA, bioelectrical impedance; D2O, deuterium dilution test; FMI, fat mass index; FFMI, fat-free mass index; MUAC, mid-upper arm circumference; SAM, severe acute malnutrition.

* Imputed data had a sample size for all variables of 100 children with prior SAM and eighty-five controls. In the original data, four children from each group were missing BIA data, two SAM children were missing D2O data and one control child was missing grip strength data.

† Marginal means controlling for age and sex; additional control for HIV exposure or infection and socio-economic tercile changed these only minimally.

‡ Multivariable coefficients represent the difference between children who experienced SAM and those who did not, adjusted for age, sex, HIV exposure or infection and socio-economic tercile.

§ Analyses conducted with log10-transformed variables; marginal means and confidence intervals from back-transformed results are presented and coefficients are from log analyses.

**Table 3.** Association of prior exposure to SAM with clinical variables strength using data from multiple imputation* (Numbers; 95 % confidence intervals)

Outcome	*n*	Imputed marginal means†	95 % CI	Difference adjusted for age and sex	95 % CI	*P*	Multivariable coefficient‡	95 % CI	*P*
Hb (g/l)									
No SAM	85	113	108 117	–6·7	–13·3, −0·0	0·049	–7·8	–14·7, −0·8	0·03
SAM	100	106	102, 110						
HbA1c (%)									
No SAM	85	5·7	5·5, 5·8	0·2	–0·1, 0·6	0·16	0·2	–0·1, 0·6	0·18
SAM	100	5·9	5·6, 6·2						
Systolic blood pressure (mmHg)									
No SAM	85	92	90, 94						
SAM	100	95	93, 97	3·1	0·2, 5·9	0·04	3·4	0·4, 6·4	0·03
Diastolic blood pressure (mmHg)									
No SAM	85	62	60, 64						
SAM	100	62	60, 84	–0·1	–2·8, 2·5	0·91	–0·1	–2·7, 2·6	0·96
Blood lipids									
triglycerides (nmol/l)									
No SAM	85	0·89	0·80, 0·98	–0·09	–0·22, 0·03	0·15	–0·10	–0·23, 0·02	0·09
SAM	100	0·80	0·72, 0·87						
Cholesterol (nmol/l)									
No SAM	85	3·73	3·58, 3·89	–0·09	–0·31, 0·13	0·42	–0·14	–0·36, 0·09	0·22
SAM	100	3·65	3·51, 3·78						
HDL (nmol/l)									
No SAM	85	1·12	1·07, 1·18	–0·05	–0·13, 0·03	0·21	–0·05	–0·13, 0·02	0·19
SAM	100	1·07	1·03, 1·12						
LDL (nmol/l)									
No SAM	85	2·27	2·14, 2·39	–0·00	–0·18, 0·18	0·98	–0·04	–0·23, 0·14	0·65
SAM	100	2·26	2·15, 2·37						

SAM, severe acute malnutrition.

* Imputed data had a sample size for all variables of 100 children with prior SAM and eighty-five controls. In the original data, four prior SAM children and six control children were missing Hb data, twenty-six prior SAM children and fifteen control children were missing HbA1c data, one child from each group was missing blood pressure data and five control children were missing blood lipid data.

† Marginal means controlling for age and sex; additional control for HIV exposure or infection and socio-economic tercile changed these only minimally.

‡ Multivariable coefficients represent the difference between children who experienced SAM and those who did not, adjusted for age, sex, HIV exposure or infection, and socio-economic tercile.

Anaemia was common in both groups: fifty-three (55 %) moderate and ten (10 %) severe anaemia among the prior SAM children and thirty-five (44 %) moderate and four (5 %) severe anaemia among the control children. Twelve (16 %) of children in the prior SAM group and four (6 %) of the control group had HbA1c > 6·5 %, suggesting diabetes. Two children, both from the prior SAM group, had both severe anaemia and high HbA1c. Blood lipid concentrations were normal for this age of children^(19)^. Prior SAM was associated with lower Hb, both when adjusted for age and sex and when fully adjusted, but was not significantly associated with other biochemical variables (Table 3).

Systolic, but not diastolic, blood was significantly higher in the prior SAM than in the control children (Table 3). When compared with age- and sex-specific normal levels^(20)^, five prior SAM children and five control children had systolic, diastolic or both blood pressure measurements over the 95th percentile which, on repeat measurements which we did not have, would suggest hypertension.

## Discussion

Children who were hospitalised for SAM when under 2 years old had, by age 7–12 years, similar anthropometric and body composition measures to neighbourhood controls who had not suffered SAM. Though the prior SAM children were slightly smaller, as indicated by negative coefficients for most comparisons, few growth differences from control children, only for hip circumference, suprailiac skinfold and FFMI by D2O, remained significant after adjustment for HIV and SES. The children who had experienced SAM had slightly lower Hb and higher systolic blood pressure than the controls but no differences in blood lipid levels. Overall there was little evidence that, by age 7–12 years, prior SAM put the children at greater risk than their neighbours for either undernutrition or overweight, excess fat and NCD.

There is limited evidence as to whether severe postnatal malnutrition during early childhood has long-term, post-recovery, effects on growth, nutrition and health^(21)^. Much of the data come from follow-up studies of anorexia nervosa^(7)^ or famine^(8,9)^ in high- or middle-income countries where, after the resolution of the disease or famine, people generally have access to a good diet and live in clean environments. These studies may not reflect the situation of children who develop SAM in poor households in low-income countries. A recent systematic review investigated cardiometabolic risks after long-term follow-up of people who experienced childhood SAM^(21)^; the authors found fourteen studies and concluded there was evidence for long-term increased risks of chronic diseases in survivors of SAM although there were issues of study quality such as small sample sizes and risk of bias^(21)^. Malawian children who experienced early malnutrition had ongoing anthropometric deficits when aged about 9 years, similar to our cohort’s age, but no evidence of altered blood lipids or HbA1c compared with neighbourhood controls^(22)^. It is notable that these Malawian children had considerably lower height *Z* score than our Zambian cohort, suggesting an underlying population difference in nutritional status^(22)^. A recent large study following up young adults who experienced malnutrition in early childhood found continuing deficits, compared with neighbourhood controls, in height but not in BMI or blood lipids^(23,24)^. Additional studies with adequate sample sizes are needed.

A major concern in all studies following up children who experienced SAM is that, because SAM mortality is high both during hospitalisation and post-discharge, especially in areas of high HIV prevalence^(2,22,23,25)^, there is a large survivor bias at follow-up. This concern is not only for cohort studies but also for the retrospective ones studying young adults hospitalised for SAM as children^(23)^, or middle-aged people who experienced famine as children^(8,9)^. Furthermore, in cohort follow-up studies, the poorest families who often live in informal or transient accommodation and do not always have mobile phones may be the hardest to follow up several years after discharge from hospital. In our study, a large proportion of hospital records for children with SAM were missing current contact information. Loss of the poorest or sickest children will likely reduce any differences from not-previously malnourished children.

Anaemia, both moderate and severe, was common among both previously malnourished and control children. We do not have diet data from the participants, but their low SES suggests that their diets are likely to be limited in foods rich in key anaemia-related micronutrients such as iron, folate and vitamin B_12_. They are also at risk of helminth infections, which may be largely asymptomatic since they were not reported, which can deplete iron stores, and inflammation which can result in anaemia of chronic disease. On the other hand, high HbA1c was quite common which provides some evidence of a double burden of anaemia and chronic diseases. The children were too young to yet exhibit signs of diabetes, but it will be important to monitor them over time.

It is not surprising to find imperfect agreement between different methods for measuring body composition since they have different underlying assumptions; we elected to analyse the different measures separately since, if they showed similar associations with prior malnutrition, this would strengthen confidence in the association^(26)^. Although there were differences in whether or not the different measures of body fat – the three skinfolds and FMI by BIA or D2O – differed between SAM and control children with *P* < 0·05, they all showed very similar pictures with the SAM children having slightly lower body fat. However, FFMI by D2O appeared lower in the prior SAM children whereas FFMI by BIA, though lower in the SAM children, was not different between the groups. Furthermore, we chose to present the body composition results as indices, rather than in kg, to account for differences in height which greatly influences FFM. However, the small and not significant differences in height of the two groups, if considered together with the difference in FFMI by D2O, might have suggested a more clinically important reduction in FFM.

The strengths of the study include its detailed anthropometry, body composition, and blood lipid and other biomarker data and its use of multiple imputation to account for missing data, most of which was missing completely at random due to problems with equipment or supplies. There is evidence that both body composition and HbA1c measured in mid-childhood can predict adult risk of high body fat and low FFM^(27,28)^ and long-term diabetes risk^(29)^. Limitations include the impossibility of randomising to SAM so an inability to determine causal associations, the relatively young age of the participants who are thus unlikely to have yet developed chronic diseases, the fact all children were from similar, largely low SES, neighbourhoods which reduce generalisability of the results, and the difficulty of following up children years after hospitalisation, mainly due to frequent moving house or missing contact details such as mobile phone numbers, with consequent likely selection bias. Thus, our cohort may not have been representative of the originally malnourished population, although it may have better reflected the survivors.

In conclusion, our study found no strong evidence of long-term adverse effects of prior hospitalisation with SAM. However, the SAM survivors may have been at slightly higher risk of the double burden of anaemia and abnormal glucose metabolism, and these risks should be kept under review as the children age. As with most studies of long-term follow-up of people who suffered childhood SAM, loss to follow-up for generally unknown reasons reduces the representativeness of our findings to the general population.

## Supplementary material

For supplementary material accompanying this paper visit https://doi.org/10.1017/S0007114521003457. click here to view supplementary material
