# Supplementary material for: Anthropometry, body composition and chronic disease risk factors among Zambian school-aged children who experienced severe malnutrition in early childhood
Source: Br J Nutr. 2021 Sep 6;128(3):453–60. doi: 10.1017/S0007114521003457 (PMC9340851; doi:10.1017/S0007114521003457)
Supplement: Supplementary file 1 [file S0007114521003457sup.zip › S0007114521003457sup001.docx]

**Supplementary Figure 1. Follow-up of participants previously hospitalised with severe acute malnutrition (SAM)**

Medical records found for children hospitalised for SAM 2010-2014

N=2034

Contact details available in records for children hospitalised for SAM

N=687

Children’s families contacted

N=157

Contact details out of date or incorrect

N=530

Recruited

N=100

Refused, N=30

Moved, N=9

Ineligible, N=3

Not included because planned sample size already reached, N=15

No contact details in records

N=1347
