# Supplementary material for: Anthropometry, body composition and chronic disease risk factors among Zambian school-aged children who experienced severe malnutrition in early childhood
Source: Br J Nutr. 2021 Sep 6;128(3):453–60. doi: 10.1017/S0007114521003457 (PMC9340851; doi:10.1017/S0007114521003457)
Supplement: Supplementary file 1 [file S0007114521003457sup.zip › S0007114521003457sup002.docx]

**Supplementary Table 1. Association of prior exposure to severe acute malnutrition (SAM) with anthropometry, body composition and grip strength, not using multiple imputation^1^**

| **Outcome** | **N** | **Mean (SD) or**  **median (IQR)** | **Difference adjusted for age and sex (95% CI)** | **P** | | **Multivariable coefficient (95% CI)^4^** | **P** |
| --- | --- | --- | --- | --- | --- | --- | --- |
| Height (cm) no SAM  SAM | 85  100 | 134.0 (13.6)  124.0 (7.1) | -1.02 (-3.19, 1.15) | 0.36 | -1.23 (-3.48, 1.01) | | 0.28 |
| Height-for-age Z no SAM  SAM | 85  100 | -0.64 (1.17)  -0.95 (0.98) | -0.15 (-0.50, 0.21) | 0.41 | -0.19 (-0.55, 0.18) | | 0.32 |
| Weight (kg) no SAM  SAM | 85  100 | 29.2 (8.8)  23.0 (4.0) | -1.40 (-3.03, 0.23) | 0.09 | -1.50 (-3.19, 0.19) | | 0.08 |
| Body mass index (kg/m^2^)  no SAM  SAM | 85  100 | 15.9 (2.4)  14.9 (1.5) | -0.46 (-1.08, 0.17) | 0.15 | -0.47 (-1.12, 0.17) | | 0.15 |
| Body mass index-for-age Z  no SAM  SAM | 85  100 | -0.58 (1.08)  -0.77 (0.94) | -0.22 (-0.55, 0.12) | 0.16 | -0.19 (-0.54, 0.15) | | 0.27 |
| MUAC (cm) no SAM  SAM | 85  100 | 19.2 (2.8)  17.6 (1.5) | -0. 5 (-1.1, 0.2) | 0.16 | -0. 5 (-1.1, 0.2) | | 0.17 |
| Hip circumference (cm) no SAM  SAM | 85  100 | 69.5 (10.4)  62.0 (4.9) | -2.3 (-4.4, -0.2) | 0.03 | -2.4 (-4.6, -0.2) | | 0.03 |
| Waist circumference (cm)  no SAM  SAM | 85  100 | 56.8 (5.9)  53.4 (3.2) | -0.8 (-2.1, 0.5) | 0.25 | -0.9 (-2.2, 0.5) | | 0.21 |
| Triceps skinfold (mm)^3^ no SAM  SAM | 85  100 | 7.8 (6.2, 9.7)  6.8 (5.8, 8.1) | -0.03 (-0.07, 0.01) | 0.11 | -0.03 (-0.07, 0.01) | | 0.12 |
| Subscapular skinfold (mm)^3^  no SAM  SAM | 85  100 | 6.2 (5.2, 7.3)  5.2 (4.8, 6.3) | -0.04 (-0.07, -0.00) | 0.05 | -0.03 (-0.07, 0.00) | | 0.07 |
| Suprailiac skinfold (mm) ^3^  no SAM  SAM | 85  100 | 5.1 (4.0, 6.3)  4.0 (3.8, 5.0) | -0.05 (-0.09, -0.00) | 0.03 | -0.04 (-0.09, 0.00) | | 0.05 |
| FMI by BIA (kg/m^2^) ^3^ no SAM  SAM | 81  95 | 2.8 (2.4, 3.4)  2.5 (2.3, 2.8) | -0.04 (-0.07, -0.0) | 0.04 | -0.03 (-0.07, 0.0) | | 0.08 |
| FMI by D2O (kg/m^2^) ^3^ no SAM  SAM | 84  97 | 2.7 (1.8, 3.5)  2.5 (1.8, 3.1) | 0.02 (-0.04, 0.07) | 0.53 | 0.02 (-0.04, 0.08) | | 0.50 |
| FFMI by BIA (kg/m^2^) no SAM  SAM | 81  95 | 12.7 (1.2)  12.1 (1.0) | -0.22 (-0.57, 0.13) | 0.22 | -0.15 (-0.51, 0.22) | | 0.44 |
| FFMI by D2O (kg/m^2^) no SAM  SAM | 84  97 | 12.9 (1.2)  12.2 (1.3) | -0.48 (-0.89, -0.08) | 0.02 | -0.39 (-0.80, 0.03) | | 0.07 |
| Grip strength (kg) no SAM  SAM | 84  100 | 14.4 (4.1)  11.3 (2.8) | -0.8 (-1.7, 0.1) | 0.08 | -0.7 (-1.6, 0.2) | | 0.11 |

^1^ BIA=bioelectrical impedance; D2O=deuterium dilution test; FMI=fat mass index; FFMI=fat-free mass index; MUAC=mid-upper arm circumference; SAM=severe acute malnutrition

^2^ Multivariable coefficients represent the difference between children who experienced SAM and those who did not, adjusted for age, sex, HIV exposure or infection, and socioeconomic tercile.

^3^ Analyses conducted with log10-transformed variables; medians and inter-quartile ranges are presented and coefficients are from log analyses.

**Supplementary Table 2. Association of prior exposure to severe acute malnutrition (SAM) with clinical variables, not with multiple imputation^1^**

| **Outcome** | **N** | **Mean (SD)** | **Difference adjusted for age and sex (95% CI)** | **P** | | **Multivariable coefficient (95% CI)^4^** | **P** |
| --- | --- | --- | --- | --- | --- | --- | --- |
| Hemoglobin (g/L) no SAM  SAM | 79  96 | 113 (19)  106 (20) | -6.5 (-13, 0) | 0.056 | -7.8 (-15, -0.1) | | 0.03 |
| HbA1c (%) no SAM  SAM | 70  74 | 5.6 (0.6)  5.8 (1.1) | 0.3 (-0.0, 0.6) | 0.09 | 0.3 (-0.1, 0.6) | | 0.13 |
| Systolic blood pressure (mmHg)  no SAM  SAM | 84  99 | 94 (10)  94 (9) | 2.8 (-0.2, 5.9) | 0.07 | 3.3 (0.1, 6.5) | | 0.046 |
| Diastolic blood pressure (mmHg) no SAM  SAM | 84  99 | 63 (8)  61 (8) | -0.2 (-2.8, 2.5) | 0.90 | -0.4 (-3.2, 2.3) | | 0.90 |
| Blood lipids |  |  |  |  |  | |  |
| Triglycerides (nmol/L) no SAM  SAM | 80  100 | 0.90 (0.43)  0.79 (0.35) | -0.09 (-0.22, 0.04) | 0.19 | -0.11 (-0.24, 0.03) | | 0.12 |
| Cholesterol (nmol/L) no SAM  SAM | 80  100 | 3.77 (0.64)  3.62 (0.65) | -0.09 (-0.31, 0.13) | 0.41 | -0.14 (-0.37, 0.08) | | 0.21 |
| HDL (nmol/L) no SAM  SAM | 80  100 | 1.13 (0.22)  1.07 (0.21) | -0.05 (-0.12, 0.02) | 0.15 | -0.05 (-0.13, 0.02) | | 0.16 |
| LDL (nmol/L) no SAM  SAM | 80  100 | 2.29 (0.54)  2.25 (0.52) | 0.0 (-0.18, 0.18) | 0.97 | -0.04 (-0.23, 0.14) | | 0.65 |

^1^ HbA1c=haemoglobin A1c; SAM=severe acute malnutrition

^2^ Multivariable coefficients represent the difference between children who experienced SAM and those who did not, adjusted for age, sex, HIV exposure or infection, and socioeconomic tercile.
